# Transcriptome Profiling of Mouse Embryonic Fibroblast Spontaneous Immortalization: A Comparative Analysis

**DOI:** 10.3390/ijms25158116

**Published:** 2024-07-25

**Authors:** Jocshan Loaiza-Moss, Ursula Braun, Michael Leitges

**Affiliations:** Division of Biomedical Sciences, Faculty of Medicine, Memorial University of Newfoundland, 300 Prince Philip Drive, St. Johns, NL A1B 3V6, Canada; jmloaizamoss@mun.ca (J.L.-M.); ubraun@mun.ca (U.B.)

**Keywords:** spontaneous cell immortalization, mouse embryonic fibroblast (MEF), RNA-seq, transcriptome profiling, gene regulatory network (GRN)

## Abstract

Cell immortalization, a hallmark of cancer development, is a process that cells can undergo on their path to carcinogenesis. Spontaneously immortalized mouse embryonic fibroblasts (MEFs) have been used for decades; however, changes in the global transcriptome during this process have been poorly described. In our research, we characterized the poly-A RNA transcriptome changes after spontaneous immortalization. To this end, differentially expressed genes (DEGs) were screened using DESeq2 and characterized by gene ontology enrichment analysis and protein–protein interaction (PPI) network analysis to identify the potential hub genes. In our study, we identified changes in the expression of genes involved in proliferation regulation, cell adhesion, immune response and transcriptional regulation in immortalized MEFs. In addition, we performed a comparative analysis with previously reported MEF immortalization data, where we propose a predicted gene regulatory network model in immortalized MEFs based on the altered expression of *Mapk11*, *Cdh1*, *Chl1*, *Zic1*, *Hoxd10* and the novel hub genes *Il6* and *Itgb2*.

## 1. Introduction

After a certain number of cell divisions, most primary mammalian somatic cells in culture enter a non-proliferative state of replicative senescence. Although senescent cells undergo a stable cell cycle arrest, these cells remain viable and undergo metabolic and gene expression changes [1,2,3]. In vivo, this process can be induced by multiple stressors such as mitochondrial dysfunction, oncogene activation, and genomic instability [4,5,6,7,8,9]. However, some cells escape senescence and acquire the ability to proliferate indefinitely, becoming immortalized [10].

Several techniques for cell immortalization have been developed including the expression of viral oncoproteins such as SV40 large T-antigen, activation of oncogenes, overexpression of telomerase reverse transcriptase (*hTERT*) or cell cycle regulators to achieve conditional immortalization [11,12,13,14]. While most techniques are induced by exogenous agents, spontaneous immortalization presents the stochastic ability of cells to overcome senescence in vivo. Studies indicate that several cell types from different mammalian models, such as mouse and chicken embryonic fibroblasts and human epithelial and rat stem cells, are capable of spontaneous immortalization in vitro [15,16,17,18,19].

Mouse embryonic fibroblasts (MEFs) are one of the primary models for spontaneous immortalization [20,21]. In MEFs, cell immortalization has been associated with changes in expression and/or mutations, genomic instability and/or epigenetic alterations. Some of the most commonly reported markers described in spontaneously immortalized proliferating MEFs include alterations in the p16^INK4a^ and p19^ARF^ pathways, such as in the loss of P53 activity and the downregulation of p21 [22,23]. Other immortalizing events such as amplification of *Mdm2* and the upregulation of *miR-21* and *miR-28*, have been reported as well [24,25,26,27]. In addition, spontaneously immortalized MEFs have been shown to exhibit chromosomal DNA damage in the form of activated DNA damage repair markers and hyperdiploidy [28], contributing to genomic instability.

Spontaneous cell immortalization represents a phenotype of clinical importance, as a feature present in a high percentage of human cancers is the acquisition of an unlimited proliferative potential [29]. The various molecular alterations that facilitate immortalization are thought to represent early genetic events in human carcinogenesis [10,30,31,32]. Considering that carcinogenesis is a multi-step process involving the accumulation of genetic and genomic alterations over time, understanding the role of spontaneous immortalization and the changes in gene expression in immortalized cells will lead to a better understanding of subsequent transformation and tumorigenesis.

Spontaneous immortalization of MEFs was first studied in 1963 [33] and has been used in functional studies for several decades. Correspondingly, spontaneously immortalized MEFs have been used to model human cancers in vitro and to study mutation landscapes relevant to cancer development [34,35]. However, very little information is available on the global changes in gene expression during the spontaneous immortalization of MEFs. Understanding these differences can help elucidate candidate biomarkers to identify immortalized cells and further aid in potentially distinguishing transformation-driving events. To investigate the changes in the transcriptome landscape in MEFs, we analyzed the transcriptomic profile of spontaneously in vitro immortalized wild-type (WT) MEFs following a modified 3T3 protocol and performed comparative analyses between our results and previously reported transcriptomic data.

## 2. Results

### 2.1. Transcriptome Changes during MEF Spontaneous Immortalization Show a Predominant Gene Downregulation Pattern

During the process of spontaneous immortalization, cells undergo significant changes in their gene expression [36,37,38,39]. To understand spontaneous cell immortalization and its role in carcinogenesis, we characterized the poly-A transcriptome changes during this process using a MEF model. We generated three immortalized MEF cell lines from three independent primary wild-type MEF clones, using a modified 3T3 protocol (see “Materials and Methods—Section 2.1” for details), and collected samples at two time points for RNA-seq: pre- (primary) and post-immortalization.

**Figure 1:**
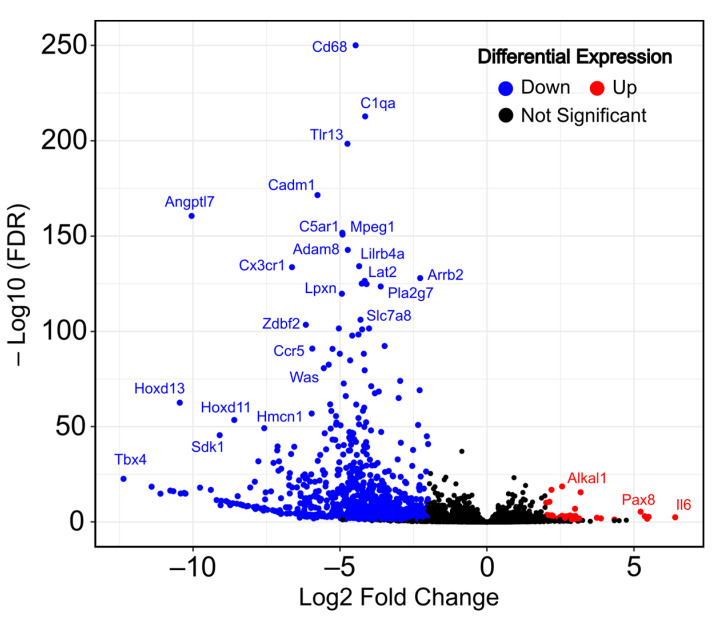
Transcriptomic changes during mouse embryonic fibroblast (MEF) immortalization (immortalized vs. primary). Volcano plot shows the up- and downregulated differentially expressed genes in RNA-seq data. Dots in blue (left) indicate downregulated DEGs and dots in red (right) indicate upregulated DEGs.

After the immortalization process, we identified a total of 1096 differentially expressed genes when applying standard thresholds of a |Log2FC| ≥ 1 and an adjusted *p*-value (FDR) < 0.01. As shown in the volcano plot (Figure 1), the transcriptomic changes show a pattern of a higher number of differentially downregulated genes than upregulated genes in immortalized MEFs. Specifically, we identified 1001 downregulated genes (shown in blue), with genes such as *Cd68*, *C1qa* and *Tlr13* as the most significant, versus 95 upregulated genes (shown in red), including *Alklal1*, *Pax8* and *Il6*. A complete list of all total up- and downregulated genes can be found in Supplementary Table S1.

**Figure 2:**
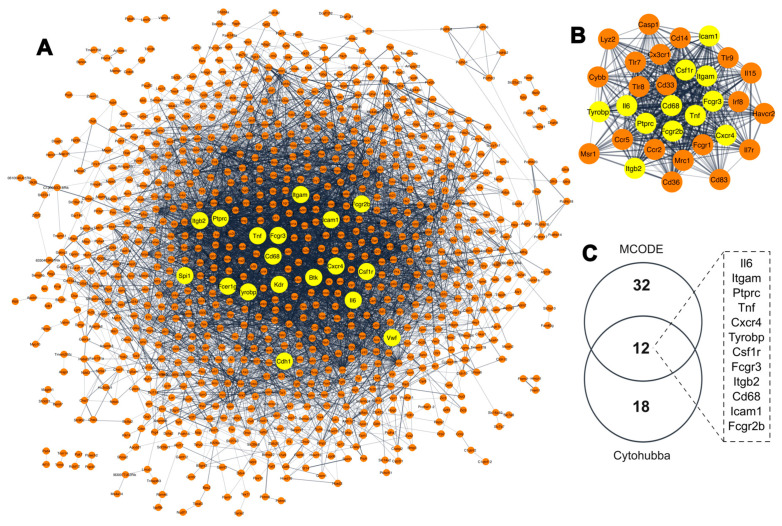
Identification of gene hubs associated with spontaneous immortalization of MEFs. (**A**) Protein–protein interaction (PPI) network generated using the STRING database. Node color: yellow indicates the hub genes, and orange nodes represent DEGs. (**B**) The highest score clustering module with 32 hub genes generated by MCODE from the PPI network; yellow nodes indicate the hub genes overlapping with the hub genes identified by Cytohubba. (**C**) Venn diagram of 12 overlapping gene hubs identifies by Cytohubba and MCODE clustering algorithms for String.

Considering the high number of DEGs identified during the immortalization process and aiming to further explore the relationship between them, we performed a protein–protein interaction (PPI) analysis using the STRING database, where we constructed a network consisting of 1083 protein nodes and 6135 interaction edges (Figure 2A). Subsequently, we identified the hub genes, i.e., genes with a high number of interactions and predicted to be of high relevance to multiple biological pathways [40,41,42]. Using the 11 topological analysis methods of the Cytohubba hub identifying algorithm, we extracted the top 20 hub genes from the main PPI network for each method. Eighteen genes were found in the intersection of at least five of these methods (shown as yellow nodes in Figure 2A). We further verified the identified hub genes using the molecular complex detection (MCODE) cluster module identification algorithm, where we obtained a clustering module with 32 hub gene nodes (highest scoring module from the PPI network of all DEGs; MCODE score: 28.645) (Figure 2B).

Comparing the two algorithms, we identified 12 overlapping hub genes between MCODE and Cytohubba, namely, integrin subunit alpha M (*Itgam*), protein tyrosine phosphatase receptor type C (*Ptprc*), Tumor necrosis factor (*Tnf*), CXC motif chemokine receptor 4 (*Cxcr4*), TYRO protein tyrosine kinase-binding protein (*Tyrobp*), Colony stimulating factor 1 receptor (*Csf1r*), Low affinity immunoglobulin gamma Fc region receptor III (*Fcgr3*), Integrin subunit beta 2 (*Itgb2*), Cluster of differentiation 68 (*Cd68*), Intercellular Adhesion Molecule 1 (*Icam1*), Interleukin 6 (*Il6*) and Low affinity immunoglobulin gamma Fc region receptor II (*Fcgr2b*), which were then considered to be the immortalization-related hub genes (Figure 2C). Most of these hub genes were downregulated during immortalization, except for *Il6*, which was upregulated. The complete list of the candidate hub genes for each Cytohubba method is shown in Supplementary Table S2.

### 2.2. Spontaneous MEF Immortalization is Associated with the Upregulation of Genes Involved in Epithelial Cell Proliferation and the Downregulation of Cell Adhesion and Immune Response

To further elucidate the functions and pathways associated with the identified DEGs, we performed a gene ontology (GO) functional enrichment analysis for biological processes and molecular functions, as well as a Kyoto Encyclopedia of Genes and Genomes (KEGG) pathway analysis: (A) an over-representation analysis (ORA) for both upregulated and downregulated genes individually and (B) a full gene set enrichment analysis (GSEA).

**Figure 3:**
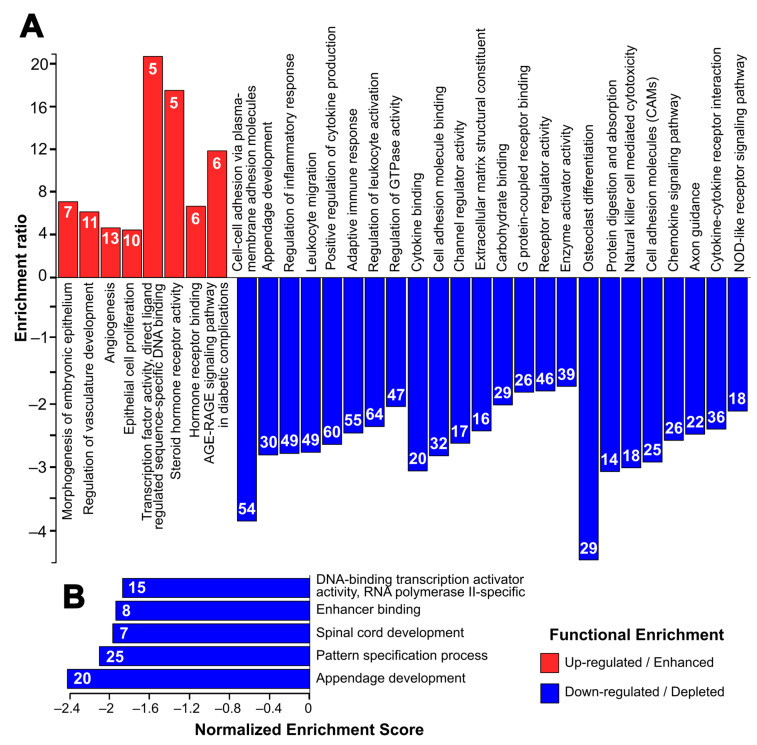
Top enriched gene ontology terms (biological processes and molecular functions) and KEGG pathways generated by Webgesalt 2019. (**A**) Terms associated with upregulated and downregulated DEGs in over-representation analysis, and (**B**) terms associated with DEGs in gene set enrichment analysis (GSEA). Color: Red (Upregulated) and Blue (Downregulated). The number inside the bars indicates the number of genes within the DEGs associated with the GO or KEGG term.

Biological processes enriched among the upregulated genes include events related to angiogenesis and vasculature development (Figure 3A), highlighted by genes such as *Pax8*, *Aldh1a1* and *Grem1*. The regulation of epithelial cell proliferation is also an upregulated biological process, including *Mapk11*, *Dusp10*, *Il6* and *Vegfa* genes. At the same time, the upregulated genes also show enrichment of several molecular functions: (a) transcription factor activity, where some of the genes include members of nuclear receptor families such as *Nr2f1*, *Nr4a1* and *Ar*, and (b) hormone receptor activity with genes such as *Vdr*, *Nrip1* and *Gnao1*.

Among the downregulated genes, the most significant biological processes depleted include cell–cell adhesion via plasma membrane adhesion molecules, circumscribed by gene families such as cadherins and protocadherins, including *Cdh1*, *Cdh19*, *Pcdh10* and *Pcdh17*. Immune response-related processes, including leukocyte migration and activation, and inflammatory response, are also downregulated, highlighting genes encoding for NLR proteins (*Nlr1x* and *Nlrp3*), and immunoglobulins (*Fcgr1*, *Fcgr2b* and *Fcgr3*). These downregulated genes possess molecular functions such as cell adhesion molecule binding, extracellular matrix structural component and receptor regulator activity. Overall, spontaneous immortalization downregulates genes associated with multiple KEGG pathways including: (a) osteoclast differentiation, as evidenced by decreased expression of signal-regulatory proteins, such as *Sirpa*, *Sirpb1a* and *Sirpb1b*, and tumor necrosis factor-associated proteins (*Tnf* and *Tnfrsf11a*); (b) cell adhesion, including several of the hub genes such as *Icam1*, *Itgb2*, *Itgam*, *Ptprc*; (c) the PI3K-Akt pathway (including *Pik2cg*); and (d) the B-cell receptor and immune response-regulating pathways mediated by gene families such as colony-stimulating factors, complement components and immunoglobulins. At the same time, GSEA shows a global downregulation of transcription factor activity and developmental processes such as appendage development and pattern specification (Figure 3B). The list of the top 10 terms and their associated genes generated by Webgesalt and clusterProfiler is provided in Supplementary Table S3a,b.

### 2.3. Comparative Analysis Shows a Positive Correlation between Gene Expression Levels for Both Immortalization Statuses between Different Spontaneous MEF Immortalization Studies, Revealing Novel Genes Differentially Expressed during this Process

To examine the similarities and differences between a previously described immortalization study and our current analysis, we recovered the list of DEGs reported by Tommasi et al. [43] and accessed their publicly available microarray dataset to compare gene expression of immortalized and primary MEFs (GSE39034) and performed a reanalysis of the data following a limma differential expression analysis pipeline [44]. We then performed a comparative global gene expression analysis between our RNA-seq data and their microarray data.

**Figure 4:**
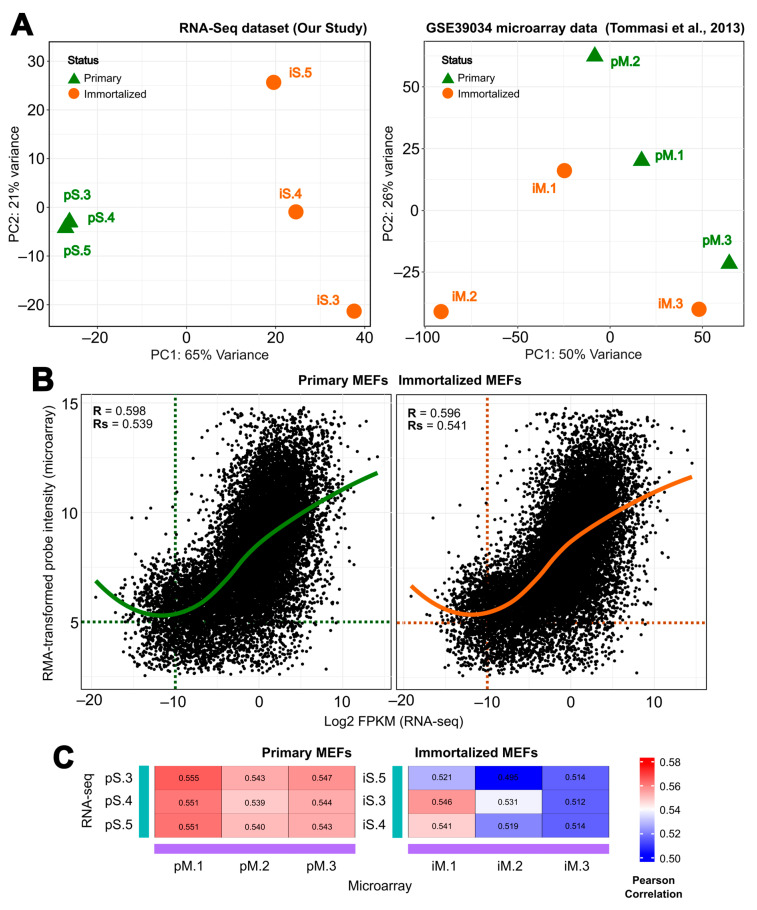
Comparison of normalized levels of gene expression during immortalization of mouse embryonic fibroblasts (MEFs) (immortalized vs. primary) between our RNA-seq and Tommasi et al. [43] microarray data. (**A**) The left panel shows the principal component analysis (PCA) of our RNA-seq data based on log2 transformation of gene expression counts derived from DESeq2 (three biological samples per state) and the right panel shows PCA of GSE39034 microarray data normalized by RMA. Green triangles indicate primary MEF samples and orange dots indicate immortalized MEF samples. Samples with the same end number follow the same embryo before and after immortalization (i.e.,—pS.3 and iS.3). (**B**) Correlation between the RMA-transformed microarray probe intensities and the log2-transformed RNA-seq FPKMs. Each dot represents the average values for each gene from all the biological replicates. Pearson’s correlation coefficient (R) is indicated for each comparison: the left panel shows a correlation for primary MEFs (green), and the right panel shows a correlation for immortalized MEFs (orange). (**C**) Sample-to-sample correlation comparison between our immortalization (RNA-seq) and Tommasi et al. [43] (microarray). The R values are plotted as a heatmap, where blue color represents lower R values, while red represents the higher R values. The left panel shows the correlation for primary MEFs, and the right panel shows the correlation for immortalized MEFs.

First, a principal component analysis (PCA) was performed on both the RNA-seq and the microarray data. In the case of our RNA-seq data, the first and second principal components (PC1 and PC2) accounted for 65 and 21% of the variability in the RNA-seq dataset, respectively, while the evaluation of the microarray dataset showed a lower degree of separation (50 and 26%) (Figure 4A). A clear separation of immortalized samples from their corresponding primary samples was observed in our immortalization (RNA-seq). However, the GSE39034 microarray data contain more noise within the samples, as they do not cluster as tightly, resulting in immortalized samples not segregating from their respective primary counterpart samples.

In addition, to assess similarities in global gene expression between both studies, we performed individual comparative analyses for both primary and immortalized samples by Pearson correlation between the RMA-transformed probe intensity and the log2-transformed FPKMs for all matching screened genes for both the microarray and the RNA-seq data. We found that gene expression was positively correlated between the studies for both pMEFs and iMEFs (Figure 4B). Specifically, for global expression, pMEFs correlated with an R-value (R) of 0.598 and iMEFs with an R of 0.596, while for gene expression of signal genes (genes with an average RMA-transformed probe intensity > 5 and a log2-transformed FPKMs > −10), pMEFs correlated with an R-value = 0.539 and iMEFs with R = 0.541. This positive correlation trend is consistent across all samples (Figure 4C), as observed in the sample-to-sample heatmaps with R values ranging from 0.539 to 0.555 for pMEFs. However, the sample-to-sample correlation between immortalized samples is slightly lower than between primary samples, with R values ranging from 0.495 to 0.546.

**Figure 5:**
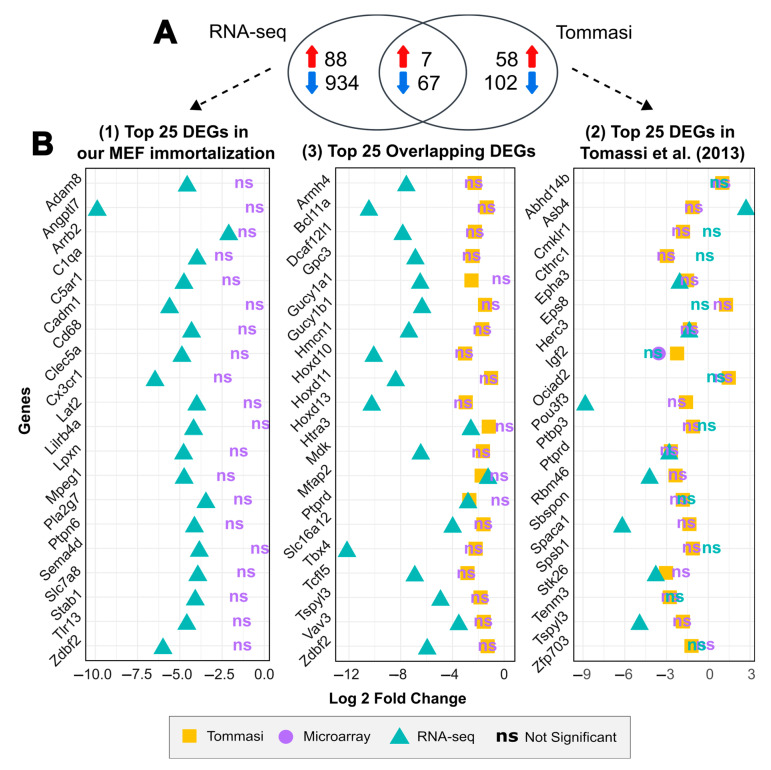
Comparison of the differentially expressed genes between different MEF spontaneous immortalization studies. (**A**) Venn diagram of 74 overlapping DEGs between our immortalization and reported by Tommasi et al. [43]. (**B**) Comparative analysis of the statistical significance of differential expression for the top 25 most differentially expressed genes found in our study (left panel), the top 25 most differentially expressed genes reported by Tommasi et al. (right panel) and the top 25 most significant overlapping DEGs between both studies (middle panel) using three MEF spontaneous immortalization datasets. The comparison shows the following: 1. GSE39034 microarray data—results reported by Tommasi et al. (yellow square); 2. GSE39034 microarray data—reanalysis (purple dot); and 3. our RNA-seq data (blue triangle); ns represents genes not statistically significant (−1 > log 2-fold change < 1 or/and *p*-adjusted (FDR) > 0.05).

Furthermore, to compare gene expression between the two immortalization studies, we performed a Venn diagram to identify common and variable differentially expressed genes identified in our immortalization assay and those reported by Tommasi et al. [43] (Figure 5A). Interestingly, we found an overlap of 74 genes being deregulated in both sets, 7 of which were upregulated and 67 were downregulated, while an additional 1022 genes were shown to be deregulated only in our dataset compared to 160 genes published in Tommasi et al. [43]. Of note, none of the gene hubs identified in our immortalization were shown to be differentially expressed in the previous study. The complete list of the common DEGs found in both studies is provided in Supplementary Table S4.

In the following, we compared the top 25 most significant DEGs identified: (1) in our immortalization, (2) by Tommasi et al., and (3) as overlapping genes between both studies. To accomplish this, we used three datasets: (a) our RNA-seq data, (b) the results reported by Tommasi et al., and (c) a reanalysis of the GSE39034 microarray data (Figure 5B) (see “Materials and Methods—Section 4.5” for details), using a threshold of |Log2FC| ≥ 1 and an FDR < 0.05. Surprisingly, none of the top 25 DEGs identified in our immortalization dataset were neither reported as differentially expressed after immortalization by Tommasi et al. nor are to be shown differentially expressed in the reanalysis of the GSE39034 data (shown as purple ‘ns’ in Figure 5B—left panel). However, among the top 25 DEGs reported by Tommasi et al. (shown as yellow squares in Figure 5B—right panel), eight genes are also identified as differentially expressed in our immortalization: *Epha3*, *Herc3*, *Pou3f3*, *Ptprd*, *Rbm46*, *Spaca1*, *Stk26* and *Tspyl3*, all of which are downregulated in both studies, and *Asb4*, which shows an opposite pattern between studies.

**Figure 6:**
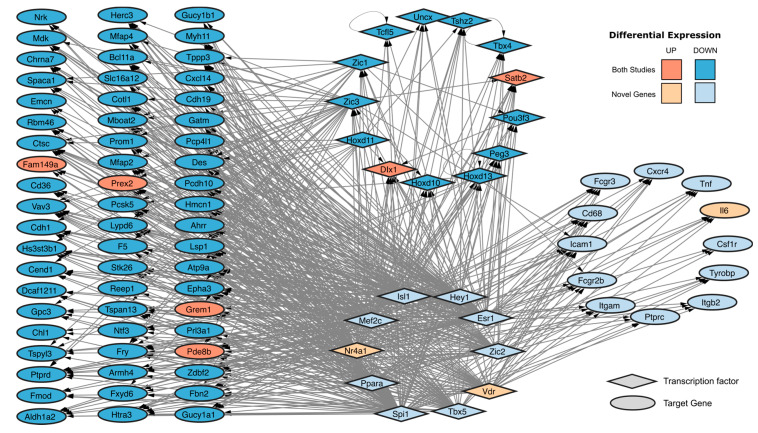
Predicted TF-target gene regulatory network on spontaneously immortalized MEFs. Shape: Diamond (Transcription factor) and Ellipse (Target gene). Color: Orange (Upregulated) and Blue (Downregulated). Opacity: Strong (identified in both studies) and weak (novel genes identified in our immortalization).

Finally, we integrated the common DEGs and our newly identified hub genes into a spontaneous immortalization-associated gene regulatory network. To accomplish this, we first identified the transcription factors (TFs) in the intersection of our DEGs with the lists of reported TFs [45,46], resulting in 82 TFs differentially expressed, 13 of which are found in both studies, namely *Dlx1*, *Hoxd10*, *Hoxd11*, *Hoxd13*, *Peg3*, *Pou3f3*, *Satb2*, *Tcfl5*, *Tshz2*, *Uncx*, *Zic1* and *Zic3*. We then extracted the predicted TF-target gene interactions using the TFLink and TRRUST databases and constructed a gene regulatory network with the common DEGs, the novel hub genes and the top 10 TFs with the highest number of interactions. We identified *Zic3*, *Hoxd11* and *Zic1* as the top three common TFs with the highest number of predicted targets, and *Spi1*, *Esr1* and *Mef2c* as the top three newly identified DE TFs with the highest number of predicted targets within the common DEGs and our newly identified hub genes (Figure 6).

## 3. Discussion

Cell immortalization is a process associated with the complex multistep development of cancer that leads to an indefinite cell proliferation phenotype by facilitating the accumulation of oncogenic alterations [47]. Currently, within the array of molecular techniques to induce and study cell immortalization, MEFs represent a well-established system model. Considering that changes in global expression during this process in MEFs are poorly described in MEFs, through this research, we aimed to explore the transcriptome landscape changes that govern immortalized MEFs after spontaneous immortalization to further unravel key aspects underlying its role in transformation and carcinogenesis.

To become spontaneously immortalized, cells acquire several genetic and epigenetic changes in cell cycle regulation [43,47], allowing for the reactivation of cell proliferation. Therefore, cell characteristics are expected to differ between immortalized and primary stages. In the case of MEFs, our study demonstrates that the multifactorial spontaneous immortalization process is accompanied by dysregulation of events related to cell proliferation regulation, cell adhesion and immune response signaling modulation, as well as transcriptional pattern variations.

The transcriptome changes in our study suggest that multiple aspects can be considered in the regulation of cell proliferation after spontaneous immortalization. In our study, we demonstrate enhanced epithelial cell proliferation via the upregulation of some members of the p38 MAPK signaling pathway, such as *Mapk11* and *Dusp10*. Mapk11 has been reported as a cell proliferation-promoting oncogene upregulated in hepatocarcinoma (HCC) and colorectal cancer (CRC), among others [48,49,50]. A previous study on MEF spontaneous immortalization [43] also showed the upregulation of *Mapk11* by hypermethylation of its regulatory CpG islands, thus reinforcing the relevance of this gene in the potential regulation of proliferation in immortalized MEFs. In the case of *Dusp10*, while its role in cancer has been shown to be mixed, Jimenez-Martinez et al. [51] report that in most cancer tissues, *DUSP10* expression levels are higher than in the corresponding normal tissue. This correlates with studies highlighting its expression as tumorigenic, as it is associated with increased cell growth in CRC [52,53].

In addition, several of the DEGs from our study may be associated with immortalization-related processes, such as (1) the transcription factor *Pax8*, which interacts with E2f1 and Rb to modulate cell proliferative responses and maintain Tert expression [54,55], suggesting an important role in spontaneous immortalization; and (2) the growth factor *Vegfa*, whose overexpression has been associated with spontaneous immortalization of murine fibroblasts [56]. In parallel, we report the downregulation of several multiple negative regulators of proliferation including *Pik3cg*, a Pi3k catalytic subunit, *Cadm1*, a cell adhesion molecule, and *Arrb2*, a G protein-coupled receptor adaptor protein. All of these have been shown to inhibit cell growth when overexpressed in cancer cells while promoting proliferation when knocked down by various approaches [57,58,59,60,61]. Taken together, our study suggests that some adaptations in MEFs during the process of spontaneous immortalization include the downregulation of genes involved in the negative regulation of proliferation, in addition to the upregulation of proliferative genes in the PI3K/Akt and MAPK pathways.

Furthermore, given the heterogeneous and dynamic nature of spontaneous immortalization, one of our goals within this study was to compare our RNA-seq data with available transcriptome data focusing on the same biological process but using a different approach, to identify independent gene expression patterns for comparison. Experimental and biological factors can be expected to increase variability when comparing gene expression between studies such as: (a) the number and locations of genetic and epigenetic variations introduced during the serial passaging or culture of primary cells and the subsequent senescence, some of which may not be conserved or relevant to the immortalization process; (b) the intrinsic heterogeneity of the embryonic fibroblast source material [62] and intrinsic inter-clonal variability; and (c) the detection sensitivity associated with the different technologies used to assess immortalization, as RNA-seq provides a more sensitive platform that can detect a higher percentage of differentially expressed genes [63,64]. However, we can observe a moderate correlation in global expression for both primary and immortalized samples between both studies, suggesting that we have a high degree of conserved changes associated with the spontaneous immortalization process.

In general, our data reveal that cell adhesion represents the most downregulated process within our immortalization, highlighted by lower expression levels of cell adhesion molecules, and components of the extracellular matrix (ECM) structure, leading to alterations in ECM–receptor interactions. While these molecules exert tumor suppressive roles mainly through cell adhesion-mediated contact inhibition, it is well characterized that disruptions in cell–cell or cell–ECM adhesion can lead to altered cell proliferation, a common phenomenon in cancer [65,66,67,68]. In the context of cell immortalization, some of these changes have been demonstrated in spontaneous and induced immortalization of MEFs, as well as in distinct human cells including esophageal and Schwann cells [43,69,70,71]. Within our analysis, we highlight three conserved cell adhesion molecules that have been previously described to be involved in proliferation, Protocadherin 10 (*Pcdh10*), Cell Adhesion Molecule L1 like (*Chl1*) and E-cadherin (*Chd1*), as candidate molecules with relevant dysregulation affecting proliferation after immortalization. (1) Pcdh10 has been reported to play a critical role in cancer cell growth by negatively regulating telomerase activity through interactions with hTert, leading to impaired telomere elongation and inhibition of cell proliferation [72]. Reduced or suppressed expression associated with methylation of the Pcdh10 promoter has been described in numerous human cancers, including multiple myeloma, gastric cancer (GC), prostate cancer and cervical cancer, where restoration of expression inhibited cell growth and migration [73,74,75,76]. (2) Reduced *CHL1* expression has been reported in neuroblastoma, and breast and nasopharyngeal cancers, where *CHL1* expression has been linked to cell growth and inhibition of metastasis by suppressing the PI3K/AKT signaling pathway [77,78,79], while its overexpression significantly increases E-cadherin expression [80]. (3) Cell dissociation during the early stage of cancer cell development has been associated with reduced E-cadherin expression; reduced expression has been reported in cell dissociation during the early stage of cancer cell development [81], and its loss is associated with tumor progression and a more aggressive phenotype [82]. Interestingly, concerning E-cadherin transcriptional regulation, we identified a representative of the p38 family of MAPKs (*Mapk11*), which is upregulated in immortalized MEFs and has been reported to downregulate E-cadherin [83]. In addition, several of our newly identified hub genes after immortalization can be associated with the regulation of E-cadherin. One of them is Interlukin-6, which has been shown to promote cell proliferation and downregulate E-Cadherin expression in osteosarcoma and bladder cancer [84,85,86]. Therefore, our report of its increased expression after spontaneous immortalization suggests a potential role in regulating proliferative and adhesion-related processes. According to Zu et al. [87], Itgb2 also corresponds to a negative regulator of tumor cell proliferation and a positive regulator of E-cadherin expression, with a lower expression level reported in non-small cell lung cancer (NSCLC) tumor tissues than in physiological tissues.

Comparing our DEGs with those reported by Tommasi et al. [43], we showed that our most significant DEGs, as well as the novel hub genes, include several immune response-related genes, such as *C1qa*, *Cd68*, *Csf1r* and *Tyrobp*. Several of these immune molecules have been reported to be downregulated in MEF SV40T-induced immortalization, where they have been reported to downregulate immune-related processes, including the B-cell receptor signaling pathway and Fc gamma-mediated processes [69]. Impaired antiviral defense and innate immune response have also been reported in spontaneously immortalized MEFs [88,89]. Our data, supported by these studies, suggest an association between the decreased immune response and expression and a potential functional role after immortalization in MEFs, which we speculate could be related to a mechanism for maintaining an immortalized state, avoiding further cellular specialization or differentiation, as well as a mechanism for cell death escape.

Moreover, among the myriad of genes with conserved changes after spontaneous immortalization, we observe the downregulation of transcription factors involved in morphogenesis and cell differentiation, including *Tbx4*, *Hoxd10*, *Hoxd11*, *Hoxd13*, *Zic1* and *Zic3*. Thus, we speculate that the downregulated genes may contribute to maintaining the immortalized cellular state of MEF as well as avoid further cellular specialization or differentiation. For example, Zic1, a zinc finger TF, plays a regulatory role in the induction of cell differentiation in several cell types [90,91,92]. Considering the number of putative targets it regulates in our GRN-like, we highlight its potential in establishing and maintaining the immortalized state in MEFs. Interestingly, Zic1 has been described as a tumor suppressor in various cancers such as CRC, GC and thyroid cancer (TC) [93,94,95], through its downregulation mainly by epigenetic regulation. In addition, some studies report that ZIC1 expression induces a cell cycle arrest by blocking PI3K/Akt and MAPK pathways, resulting in the inhibition of cell proliferation and reduced migration [95,96]. This increased proliferation observed in *ZIC1*-depleted cells has been linked to the negative transcriptional regulation of E-cadherin in HCC and CRC [95,97], whereas overexpression of *ZIC1* in GC and TC upregulated E-cadherin expression [98,99]. In our study, we also identified other differentially expressed Zic family members as well (*Zic2* and *Zic3*). For Zic3, it has been reported to be hypomethylated in cryptogenic HCC [100] and a decreased expression has been identified in malignant high-grade glioma tissues when compared to the counterpart healthy tissue [101], implying a potential role during immortalization. Considering that different studies using different models have shown that Zic proteins seem to exhibit a degree of redundancy in processes related to differentiation and proliferation [102,103,104], we predict that future studies will shed light on potential roles of Zic family members during spontaneous immortalization, while our data predict a clear functional association of this transcription factor family.

Another interesting example of a downregulated TF is *Hoxd10*, which has been reported to induce growth arrest and neuronal differentiation by downregulating cell cycle-promoting genes [105]. Demethylation or overexpression of *HOXD10* has been shown to suppress proliferation and migration, and to promote apoptosis in CRC [106], which may be related to its ability to bind E-cadherin upstream sequences and positively regulate E-cadherin expression, as described in clear cell renal cell carcinoma [107]. Thus, dysregulation of *Hoxd10* can potentially cause a decrease in E-Cadherin, which is also supported in our data set.

Regarding previous reports using spontaneous immortalization of MEF, we acknowledge that several biological processes and genes have been described to be altered or involved during and after immortalization [24,28,108,109]. Considering the scope of our study, as well as the data generated in our immortalization, we have extracted and predicted cellular events and candidates with potential biological relevance in MEFs after spontaneous immortalization.

**Figure 7:**
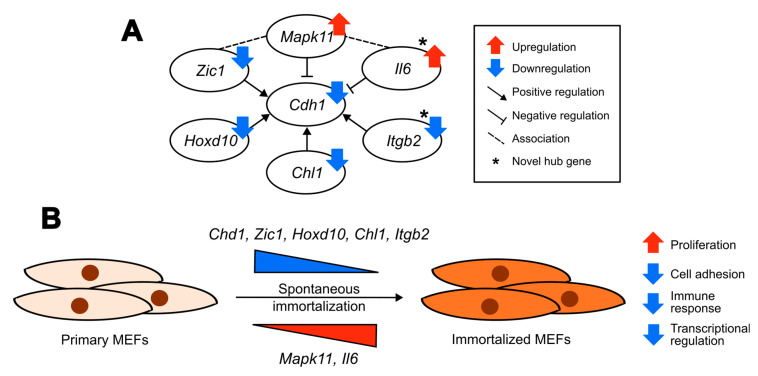
Transcriptomic profiling reveals a potential model for altered biological processes and predicted gene regulatory networks in MEFs after spontaneous immortalization. (**A**) Predicted regulatory network in MEFs after spontaneous immortalization. * indicates the novel hub genes. (**B**) Proposed model for altered biological processes, and the potential genes involved, in MEFs after spontaneous immortalization.

Based on our analysis we propose a regulatory network being involved in the process of spontaneous immortalization of MEFs, centered around *Cdh1* (Figure 7A), which is known to play a role in cell adhesion and proliferation. We predict that the upregulation of *Il6* and *Mapk11* accompanied by the downregulation of *Zic1*, *Hoxd10*, *Chl1* and *Itgb2* cause a downregulation of Cdh1 (Figure 7A). This may eventually lead to a loosening of cell contacts and a promotion of proliferation of cellular behaviors observed after immortalization, accompanied by transcriptional reprogramming and alterations in the expression of immune molecules (Figure 7B). Our proposed alterations have clinical relevance as they are found in human cancers, as previously described. According to the Human Protein Atlas database, E-cadherin is considered a potential cancer-related drug target. Similarly, both Mapk11 and Il6 are cancer-related FDA-approved drug targets and correspond to markers for an unfavorable prognosis for renal cancer [110]. Collectively, our proposed regulatory network aims to provide an additional description of cell immortalization and it represents potential markers in defining differences between primary, immortalized and transformed cells during the process of cancer development.

## 4. Materials and Methods

### 4.1. Cell Culture and Immortalization

Primary wild-type mouse embryonic fibroblasts (pMEFs) from three different embryos were previously obtained by our lab according to standard protocols [111,112,113]. pMEFs were cultured in Dulbecco’s Modified Eagle’s Medium (DMEM)—high glucose (Sigma-Aldrich, Oakville, ON, Canada, Cat#D5796-500ML) supplemented with 10% fetal calf serum (FCS) (FisherScientific, Nepean, ON, Canada, Cytiva Cat#SH3412IH345), 1X non-essential amino acids (FisherScientific, ON, Canada, Cytiva Cat#SH30238.01), 1% Pen Strep (FisherScientific, ON, Canada, Gibco Cat#15070-063) and β-mercaptoethanol (FisherScientific, ON, Canada, Gibco Cat# 21985023).

To immortalize MEFs, we used a modified NIH 3T3 protocol. Cryopreserved primary embryonic fibroblasts (passage 0) were plated on 10 cm dishes. After the first two passages, pMEFs were plated in 6-well dishes (FisherScientific, ON, Canada, Fisherbrand Cat#FB01297) and grown at 37 °C with 5% CO_2_. Cultures were passaged serially at 80% with a pass 1:3 until they reached senescence (typically 5–6 passages). Once MEFs became senescent, culture media were shifted to a MEF culture medium with 20% FCS, where the culture medium was changed twice a week. Prolonged cell culture gave rise to spontaneously immortalized cells, visualized as individual growing colonies, which then established immortalized MEF cell lines (iMEFs). After immortalization, iMEFs were expanded and grown in MEF culture medium with 10% FCS.

### 4.2. RNA Sequencing and Differential Expression Analysis

RNA sequencing was performed on pMEFs and iMEFs. The total RNA of MEFs was extracted using the AllPrep DNA/RNA Mini Kit (Qiagen, ON, Canada, Cat#80204). Poly A-enriched RNA library preparation and sequencing (Illumina NovaSeq 6000 PE100) was outsourced to Centre d’expertise et de services Génome Québec (Montreal, QC, Canada). The quality of the FASTQ files was analyzed using FASTQC (version 0.12.0) (https://www.bioinformatics.babraham.ac.uk/projects/fastqc/ (accessed on 15 December 2023)). Pair-end reads obtained from RNA-seq data were mapped to the mouse reference genome (mm10) using STAR (version 2.7.10a) software [114] with default parameters. The Sequence Alignment Map (SAM) files were converted into the Binary Alignment Map (BAM) format using Samtools (version 1.18) [115]. Gene counts were assigned based on reads uniquely mapped to annotated genes in the Ensembl Mus_musculus.GRCm38.102.gtf using the featureCounts function of Subread (version 2.0.6) [116]. Differentially expressed genes (DEGs) were acquired using the Bioconductor DESeq2 package (version 1.41.13) [117] in R software (version 4.3.2, https://www.r-project.org/ (accessed on 11 January 2024)). The RNA-seq data discussed in this publication have been deposited in the National Center for Biotechnology Information’s Gene Expression Omnibus [118] and are accessible through GEO Series accession number GSE266445.

### 4.3. Protein–Protein Interaction (PPI) Analysis and Hub Gene Identification

A PPI network of DEGs was constructed using the STRING database (version 2.0.2) and was visualized in Cytoscape (version 3.10.1) [119,120]. Based on the genes in the network, hub genes were identified through 11 topological analysis methods (MCC, DMNC, MNC, Degree, EPC, Bottleneck, EcCentricity, Closeness, Radiality, Betweenness and Stress) employing the Cytohubba Cytoscape plug-in [121]. The selected hub genes were verified by MCODE hub analysis [122], following the pipeline shown by Ma et al. [123].

### 4.4. Gene Ontology and Pathway Enrichment Analysis

Gene ontology (GO) over-representation (ORA) [124,125] and gene set enrichment analysis (GSEA), for the categories of biological process (BP) and molecular function (MF), and pathway enrichment analysis based on the Kyoto Encyclopedia of Genes and Genomes (KEGG) database [126,127] were carried out for differentially expressed mRNAs using Gene Set Analysis Toolkit WebGestalt 2019 online tool [128] and clusterProfiler (version 4.10) [129]. An FDR-corrected *p*-value < 0.05 was considered of statistical significance and redundancy reduction was considered using weighted set cover.

### 4.5. Microarray Data

GPL1261 platform data from GSE39034 accession was obtained from the GEO public database [43], where 6 arrays in two experimental groups were recovered (mouse embryonic fibroblasts, primary, passage # <6 (expression) and mouse embryonic fibroblasts, immortalized, passage # >10 (expression)). Microarray data was processed using a limma pipeline previously described in [44]. Raw CEL files were normalized using Robust multiarray analysis (RMA) and immortalized and primary expression was compared using the Bioconductor limma package (version 3.58.1) differential expression analysis [130], where an L2FC of ±1 and a FDR  <  0.05 was considered as statistically significant.

### 4.6. Microarray and RNA-seq Expression Data Correlation

Comparison between the expression levels in RNA-seq data and microarray data for both primary and immortalized MEFs were performed via Pearson correlations (R). For RNA-seq, normalized read count fragments per kilobase of transcript per million mapped reads (FPKM) were calculated and further log2-transformed using the countToFPKM package (version 1.2.0) (https://github.com/AAlhendi1707/countToFPKM (accessed on 12 February 2024)). For the microarray, the RMA-transformed expression level of each gene was collected for every probe of every gene. The correlation between the expressions of a gene in both studies was determined by the R-value. Principal component analysis (PCA) for both RNA-seq data and microarray were calculated with DESeq2 and limma package, respectively, and plotted in R to assess variance between sample groups and sample replicates. Additionally, the log2 fold change in the topmost differentially expressed genes in RNA-seq and reported by Tommasi et al. [43] were compared.

### 4.7. Transcription Factor (TF)—Target Gene Regulatory Network

Differentially expressed transcription factors after immortalization were identified in the intersection of the DEGs with two lists of transcription factors downloaded from Gifford-lab/Reprogramming Recovery GitHub repository data (n = 1374) [45] and http://bioinfo.life.hust.edu.cn/static/AnimalTFDB3/download/Mus_musculus_TF (accessed on 21 February 2024) the mouse AnimalTFDB 3.0 database (n  =  1636) [46] as described by Henze et al. [131]. TF-target gene interactions were obtained from the TFLink and TRRUST (version 2) databases [132,133] and visualized using Cytoscape.

## 5. Conclusions

Our global transcriptome characterization of primary and immortalized MEFs, in combination with the comparative analysis, shows that transcriptional changes after spontaneous immortalization have a high degree of consistency, with a tendency towards gene downregulation. We propose that immortalized cells are characterized by the upregulation of pro-proliferative genes, associated with MAPK signaling activation, including *Mapk11* and *Il6*, and the downregulation of cell adhesion molecules, such as *Itgb2*, *Chl1* and E-*cadherin*. In addition, we show that spontaneous immortalization in MEFs alters the expression of transcription factors involved in cell differentiation, as well as immune response-related molecules, potentially influencing the maintenance of the immortalized state and the escape from cell death. Further validation in MEFs, as well as in vivo screening in human models, is required to confirm the role of our proposed genes in controlling cellular processes in both immortalized MEFs and human cells, as well as their relationship to carcinogenesis. Understanding the transcriptome changes in spontaneously immortalized cells and how they differ from primary cells is a key aspect to deciphering biological markers to potentially predict and minimize cell transformation and, thus, early cancer risk.

## Data Availability

RNA-seq data presented in the study are openly available in GEO (Series GSE266445).

## Supplementary Materials

The following supporting information can be downloaded at: https://www.mdpi.com/article/10.3390/ijms25158116/s1.
